# Apparent Gains, Hidden Costs: Examining Adoption Drivers, Yield, and Profitability Outcomes of Rotavator Tillage in Wheat Systems in Nepal

**DOI:** 10.1111/1477-9552.12333

**Published:** 2019-04-29

**Authors:** Gokul P. Paudel, Vijesh V. Krishna, Andrew J. McDonald

**Keywords:** Agricultural productivity, impact heterogeneity, Nepal, propensity score matching, sustainable tillage practices, technology adoption

## Abstract

The ‘high speed’ rotavator is used for shallow tillage to create a fine tilth and incorporate crop residues, often with a single tractor pass. Rotavator tillage has spread quickly in many parts of South Asia, despite short‐term experimental trials suggesting deteriorating soil quality and crop yield penalties. Evidence of rotavator impacts on farmer fields across soil gradients and time is largely absent. From a farm household survey conducted among wheat farmers in Nepal, we estimate wheat yield and profitability outcomes for rotavator adopters and non‐adopters using propensity score matching. We find that rotavator adoption leads to inferior outcomes, despite significant cost savings for land preparation (US$ 11–15 per hectare). With rotavator adoption, farmers lose about 284–309 kg of wheat grain and about US$ 93–101 of profits on average per hectare per season, and these penalties increase with longer‐term use of the technology. Adoption of rotavator appears to be driven by the cost and time savings for land preparation. Against this backdrop, new policy and extension efforts are required that discourage rotavator use and favour more sustainable tillage technologies.

## 1 Introduction

The development of farming technologies that are environmentally sustainable and financially beneficial to farmers has become a key topic of agronomic research in the last two decades. One of the most prominent sustainable technologies is zero tillage (Derpsch *et al*., 2010; El‐Shater *et al*., 2016). Identified as one of the transformative innovations in conservation agriculture, zero tillage has the potential to enhance the adaptive capacity of farming communities to mitigate the challenges of climate change in the tropics (Harvey *et al*., 2014; Arslan *et al*., 2015; Sapkota *et al*., 2015). However, the rate of diffusion of zero tillage has remained low in many parts of South Asia (Kassam *et al*., 2014; Krishna *et al*., 2016). Traditional farmers' belief that tillage is essential for crop production has been identified as one of the major barriers to rapid diffusion of this technology (Bhan and Behera, 2014). Easy access to machines for intensive tillage, ranging from cultivator to power tiller, augments this belief. In recent years, the spread of rotavators or rotary tillers – tractor‐operated high speed tillage machinery that breaks the soil with the help of rotating ‘L’ or ‘J’‐shaped blades to create fine tilth – has been rapid in many parts of South Asia (Erenstein, 2010). However, rotavator tillage is also shown to have several negative consequences on soil quality and crop yield in experimental research trials (Tripathi *et al*., 2007; Ahmad *et al*., 2010; Schjønning and Thomsen, 2013; Guan *et al*., 2015; Sang *et al*., 2016).

If the results of these research trials hold true in farmers’ fields, diffusion of rotavator use in South Asia suggests a possibly unique case of perverse adoption, since financial viability and improvement are supposed to be the important determinants of technology adoption (Knowler and Bradshaw, 2015). There are two plausible explanations for the prevalence of rotavator tillage in South Asia. First, the negative effects of rotavator tillage could be less pronounced (or even absent) in farmers’ fields, especially in the short term. Researcher‐managed plots often differ from farmer‐managed ones with respect to production conditions and managerial efficiency. Second, adoption decisions could be made under resource constraints, and not solely based on the criteria of land productivity or profitability. In order to design technology interventions to promote resource‐conserving technologies like zero tillage, the effects of technology alternatives on yield and profits should be estimated. To the best of our knowledge, no study has been conducted so far to assess the on‐farm effects of rotavator tillage.

At present, the adoption‐impact literature is heavily biased toward documenting success stories of promising technologies (e.g., Gitonga *et al*., 2013; Shiferaw *et al*., 2014; El‐Shater *et al*., 2016; Mishra *et al*., 2016). While there exists some evidence for why certain promising technologies fail (Douthwaite *et al*., 2001), the reasons behind farmer acceptance of unsustainable technologies has rarely been the subject of analysis. We argue that documentation of the agronomic and financial effects of less‐sustainable alternatives of promising technologies also bears significant policy relevance. Here, we attempt to: (i) verify whether the effects of rotavators are negative in farmers’ fields in terms of both yield and profitability; if so, (ii) identify the rationale behind the farmer acceptance of this ‘unsustainable’ technology. Farm household data collected from the wheat‐based farming systems of Nepal are used for the empirical analysis.

Nepal is one of the least developed countries in Asia with a quarter of its population living below the absolute poverty line (NPC, 2017) and about one‐third of children under five facing acute nutritional deficiency (FAO, 2013). Food insecurity is a major challenge with more than half of Nepalese districts facing food deficit every year in the recent past (Joshi *et al*., 2012). With two‐thirds of the population engaged in agriculture, technological innovations which increase yield and profit have the potential to reduce rural poverty in Nepal. Wheat is grown on 25% of the cultivated land in the country, but the crop productivity has stagnated (MoAD, 2016). Farmer adoption of less‐sustainable practices further slows down the rate of economic growth. The insights gained from examining the field and plot conditions affected by rotavator adoption in Nepal have wider geographical significance, as diffusion of rotavator tillage is also rapid in other countries of South Asia (Krishna *et al*., 2012). In India, to date, about 600,000 rotavator units have been sold, with 50,000 new units being marketed every year (15–20% growth) by the 14 leading manufacturing companies (Rana, 2017). Experts predict an increase in the annual manufacture of rotavator units in the coming years (pers commun, R.K. Malik, International Maize and Wheat Improvement Center in 2018). Although rotavators are prevalent in other countries, as indicated by Paman *et al*. (2015) in Indonesia, Rizwan *et al*. (2017) in Pakistan, Memon *et al*. (2018) in China etc., the literature on its diffusion rate and agronomic or economic effects at the farm level has remained scant.

Background details of rotavator technology and the scope of the present study are provided in the next section. The data used for the empirical analysis are described in section 3, and the analytical framework is outlined in section 4. The empirical findings are presented in section 5, while the last section discusses these findings and concludes the study.

## 2 Background and Scope

Conventionally, tillage has been perceived as one of the most important cultivation practices that determines soil quality (Mosaddeghi *et al*., 2009; Singh *et al*., 2013), crop growth (Mosaddeghi *et al*., 2009), and short‐term and long‐term sustainability of crop production systems (Bhatt *et al*., 2016). In wheat production, tillage is found to affect physical, chemical and hydrological requirements for crop growth (Mohanty *et al*., 2007; Bazaya *et al*., 2009). However, these effects vary depending on the type of tillage practices as soil quality, water percolation and land productivity are affected differently by different tillage practices in different agro‐ecosystems (Kumar *et al*., 2013; Das *et al*., 2014). There is a growing concern about the soil productivity and wider environmental implications of conventional tillage practices, especially using iron ploughs, rotary tillers and disks (Knowler and Bradshaw, 2007), which are sometimes labeled as unsustainable (Hobbs *et al*., 2008). There are alternatives to conventional practices, such as zero‐tillage (Keil *et al*., 2015; El‐Shater *et al*., 2016) and double no‐till (Jat *et al*., 2009), that improve crop yields and save costs compared with traditional tillage (Bhan and Behera, 2014). Nonetheless, farmer adoption of these methods has been slow, perhaps reflecting a lack of initial policy and extension support (Kassam *et al*., 2014). While some unsustainable technologies are adopted quickly by farming communities due to higher opportunity cost of labour time (Low, 1993) and associated lower cost of technology adoption (Pierpaoli *et al*., 2013), the impact of such unsustainable practices on yields, costs and farm profits are rarely subjected to analysis.

The history of mechanised tillage in Nepalese agriculture dates back to early 1970s with the advent of two‐ and four‐wheel tractors (Biggs *et al*., 2011). However, diffusion has been rapid only in the last two decades. The number of farms using mechanised tillage has increased from 5% in 1995 (Takeshima *et al*., 2015) to 23% in 2016 (Takeshima, 2017a) The area under mechanised tillage has significantly increased in recent years, albeit with only a small share of farmers (<1%) owning tractors and other tillage machinery (Takeshima, 2017b). Custom hiring has become prevalent, especially in the cereal belt of Nepal Terai.

Diffusion of mechanised tillage shows high spatial heterogeneity in Nepal. While less than 8% of farms use mechanised tillage in the mountains and hills, about 46% use it in the lowland Terai region (Takeshima, 2017a). This region has been considered to have higher potential for crop intensification due to plain topography, better input access, and larger landholding size (Takeshima, 2017a,2017b). The higher concentration of four‐wheel tractors has facilitated the use of different types of tillage machineries, including tine cultivators, disc harrows, seed drills and rotavators (Biggs and Justice, 2015; Gauchan and Shrestha, 2017). On the other hand, conservation tillage practices have been less prevalent, even in the Terai region. Technologies such as direct seeded rice and zero‐ or reduced‐tillage wheat have been introduced recently but these technologies are spreading only slowly among farmers (Ghimire *et al*., 2013).

The history of rotavator tillage in Nepal is recent. Krishna *et al*. (2012) reported that the technology was introduced in many Nepal villages only after 2005, while it was prevalent in the northwestern Indian villages even in the mid‐1990s. Since Nepal and India share a porous border, farmers from Nepal Terai constantly interact with their counterparts in India. Unsurprisingly, farmers residing in the Indo‐Nepal border‐districts were the first to adopt rotavators in Nepal. Recognising the scope of an emerging market, the private sector started importing rotavators from the neighbouring countries, India and China. Tillage demonstrations were conducted by private dealers in many villages of Nepal Terai (Gauchan and Shrestha, 2017). Adoption was also driven by government subsidy programmes, governed by a farm mechanisation policy in 2014 and other mechanisation promotion policies (Takeshima, 2017a). The Government of Nepal provided up to 50% subsidy to promote farm mechanisation. There was also a reduction of tariffs for importing farm machinery, including rotavators (Gauchan and Shrestha, 2017).

During the last two decades, a number of studies have been carried out to address the agronomic effects of this new tillage option. Several research studies have shown a weak performance of rotavator tillage when compared with other tillage methods in wheat (Ahmad *et al*., 2010; Amin *et al*., 2014). Tripathi *et al*. (2007) reported inferior performance of rotavator tillage in the rice‐wheat systems in India. The negative effects are generated through increasing sub‐soil compaction or creation of a hard‐pan due to continued use of shallow tillage (Khan *et al*., 2012; Nawaz *et al*., 2013; Głab, 2014; Sang *et al*., 2016). These effects have many detrimental consequences, including lower rate of incorporation of fresh organic matter, reduced nutrient recycling and mineralisation, reduced activities of micro‐organisms, increased weed pressure, increased lodging problems, and increased wear and tear on cultivation machinery (Hamza and Anderson, 2005). Increased soil compaction also increases the bulk density, reduces pore space, impedes root growth, and requires more energy for tillage. A reduction in pore space hinders water and air movement in soil, thereby reducing water holding capacity and restricting root penetration (Ahmad *et al*., 2010). These factors ultimately lead to increased cost of production and reduced yield and profitability for farmers.

While there exist a number of studies on negative effects of rotavators, all are based solely on information from researcher‐managed field trials. While some of the socio‐economic studies have questioned the increased adoption of rotavator tillage (Erenstein, 2009, 2010), no systematic analysis has been carried out on the agronomic and economic effects of this technology in farmers’ fields. While the conventional agronomic tools such as long‐term trials have a role in documenting the intermediate and progressive impacts of tillage practices on soil characteristics and crop yields over time, they are too expensive to conduct and do not sufficiently capture technology interactions with the range of soil types and management factors. Against this backdrop, our research objective is to assess the on‐farm economic impacts of rotavator adoption in Nepal Terai, across districts with diverse socioeconomic and agro‐ecological conditions. Understanding the economic effects of rotavators is the first step toward understanding the rationale behind farmer adoption of the technology in this region.

## 3 Data

Our empirical analysis is based on the farm household data collected from wheat farmers in Nepal Terai through face‐to‐face interviews. The survey was conducted using a structured questionnaire during April–June 2016, immediately after completion of wheat harvest in the region.^2^ The questionnaire included sections on household demographics, cropping patterns, income sources, as well as wheat production technologies, inputs and practices (including tillage), outputs obtained and marketing channels.^3^ The sampling strategy consists of a purposive selection of 10 districts from Terai region and 5 sub‐districts (Village Development Committees or VDCs) from each of the selected districts. All districts and VDCs were selected based on the wheat acreage and prevalence of mechanised tillage adoption. One ward from each VDC, and 10 wheat farmers from each ward were selected randomly. This procedure yielded a sample size of 500 farm‐households. Excluding the 15 questionnaires that were incompatible for our study purpose, data from 485 households are analysed. Figure A1 in the online Appendix shows the location of sampled districts, and extent of wheat area as well as rotavator adoption in the region.

In this impact evaluation study, the ‘treatment’ group includes farm households who used rotavator tillage in the main wheat plot during the previous season of survey (winter 2015–2016). The ‘control’ group employed other tillage methods for wheat production in the same season on their main plot.^4^ It is possible that tillage decisions are different across different plots managed by a single farm household, leading to a certain degree of overlap of control and treatment groups if other plots were also considered for the analysis. To enhance precision, the effects of adoption are estimated at the plot level using the main plot, and not at the household level, as done elsewhere (Krishna and Qaim, 2012; Kassie *et al*., 2015; Mishra *et al*., 2017b). Noltze *et al*. (2012) also showed that farmer adoption can be better explained by combining plot and household characteristics while examining the impact of technology adoption in rice.

## 4 Empirical Framework

The traditional approach to evaluating the effects of technology adoption is to regress the outcome variables with an adoption dummy and a vector of farm household and plot attributes as the control variables. The underlying assumption of this approach is that adoption of technology is exogenously determined. However, there could be a number of unobserved farm household attributes (e.g. farmer's perceptions, risk aversion, managerial skills etc.) that may be correlated with both adoption and outcome variables, making observed adoption endogenous, and producing biased estimates for ordinary least squares regression analysis (Kabunga *et al*., 2012).

Researchers have proposed various estimation techniques in order to resolve the endogeneity issue (Mason *et al*., 2017). Random assignment of the treatment is inapplicable for impact assessment when data are collected after wide‐spread adoption (de Janvry *et al*., 2010). Wooldridge (2002) argues that application of panel estimators with household‐level fixed effects can control for time‐invariant heterogeneity. However, development of panel datasets is not always feasible due to time and financial constraints, and this will not control for the time‐variant heterogeneity. Selection bias can also be addressed using the Heckman two‐step method (Heckman *et al*., 1997) and instrumental variables approach. However, suitable instrumental variables are not always available (Jalan and Ravallion, 2001; Mendola, 2007).

The dataset used for this study are cross‐sectional. At the time of data collection, a significant share of households had already adopted rotavator tillage. Suitable instrumental variables were also not available from our dataset.^5^ Hence, we use matching algorithms and propensity score matching (PSM) to partly correct endogeneity, following Dehejia and Wahba (2002). The estimation of effect of technology adoption on outcome indicators using PSM is based on balancing the distribution of observed attributes of rotavator adopters and non‐adopters, and comparing differences after matching based on the similarity in their observed attributes. PSM is a non‐parametric method, and has been increasingly employed to study the effects of technology adoption in agriculture (Gitonga *et al*., 2013; Khanal *et al*., 2015; Mishra *et al*., 2016, 2017a; Mason *et al*., 2017).

To estimate the effects of rotavator tillage with PSM, we first specified the conditional probability of rotavator adoption, using a logit model to derive the propensity scores. In the second step, we matched the adopting farm households with the non‐adopters based on similarity in the propensity scores. In order to match technology adopters with non‐adopters based on their distribution of observed attributes, several algorithms have been proposed in the literature (Caliendo and Kopeinig, 2005). Following popular practice, we employed three different matching algorithms – kernel‐based matching (KBM), nearest neighbour matching (NNM), and caliper matching or radius‐based matching (RBM). Each of these matching algorithms has unique features, although robustness of estimates can be confirmed when all of them provide comparable estimates. In KBM, weighted averages of outcomes of all households in the non‐adopter group are used to construct the counterfactual. These weights are inversely associated with the distance between propensity score (Caliendo and Kopeinig, 2005). While NNM involves choosing farmers adopting and non‐adopting rotavator tillage that are closest as a matching pair in terms of propensity score (Ali and Abdulai, 2010), this is usually applied with replacement so that the control sample can be the best matched pair for more than one treated sample (Becker and Ichino, 2002). In the RBM approach, a tolerance level on maximum propensity score distance between subjects in the adopter group is estimated, which is then employed to derive subjects in the counterfactual non‐adopter group (Andam *et al*., 2008).

The main purpose of the PSM method is to balance the observed distribution of covariates across the treatment and control groups. This procedure requires a covariate balancing test after matching to ensure that there are no systematic differences in the distributions and there is an overlap of the covariates among adopters and non‐adopters (Sianesi, 2004; Lee, 2013). The results from the post‐matching two‐sample *t*‐test should not be significantly different across adoption categories for any of the covariates for meaningful comparison. The matching quality is tested by comparing pseudo *R* ^2^ and *P*‐values of the likelihood ratio of the joint insignificance obtained from the logit model before and after matching the covariates. Lower pseudo *R* ^2^ and insignificant *P*‐value of the likelihood ratio after matching would denote that the balancing property is satisfied (Sianesi, 2004). The balancing property can also be tested with mean absolute standard bias (MASB) between rotavator adopters and non‐adopters as suggested by Rosenbaum and Rubin (1985). An MASB value greater than 20% is considered too large to qualify the matching process.

A general weakness of PSM as an impact evaluation method is that the matching is based entirely on the observed characteristics. If there are certain unobserved variables that affect both the adoption decision and outcome variables (hidden biases), the resulting PSM estimates will be biased (Andam *et al*., 2008). We conducted the sensitivity analysis to identify whether the magnitude of hidden bias could alter the conclusions of the study. According to Rosenbaum (2002), the unobserved heterogeneity or hidden bias may leave visible traces in the observed data, which can be distinguished by a variety of tactics involving pattern specificity.

## 5 Results

The data contain 158 farm households (33%) adopting rotavator tillage and 327 households (67%) following other tillage practices.^6^ The descriptive statistics are presented in Table A1 in the online Appendix. The average farm size for rotavator adopters was 33% smaller than that of non‐adopters, despite no significant difference in the mean household size. Adopters and non‐adopters do not differ with respect to mean age, education, or group membership status of the household head. However, the share of households belonging to the socially marginalised castes is significantly low among the adopters.^7^ The share of household heads with agriculture as main occupation is also significantly low in the adopter group, although this is not reflected in the mean off‐farm income. A higher share of adopters owned mobile phones. The spatial patterns of technology diffusion is evident, with rotavator use being more popular in western Terai.

The difference between the rotavator adopters and non‐adopters is also noticeable in the main plot characteristics (Table A1 in the online Appendix). Farmers adopted rotavator tillage mostly in plots with silty soil, but rarely in plots with sandy soil. Adoption was significantly higher in the lowlands and in plots with irrigation facilities. Furthermore, farms with delayed harvesting activities (for the previous crop) were the ones mainly adopting rotavator tillage. These systematic differences in the mean values of observed attributes of rotavator adopters and non‐adopters potentially affect the adoption decision as well as wheat yield and profit, and hence necessitate matching and a test for selection bias.

Rotavator technology appears to be spreading rapidly in the villages where labour scarcity is relatively high, as adopters are found to be paying significantly higher wages for agricultural labourers (Table 1). While comparing the costs and returns from wheat farming, the tillage cost is significantly lower in plots prepared using rotavator tillage, and this could be one of the major drivers of adoption. The fertiliser application rates were higher in rotavator‐tilled plots, which increased the total variable costs significantly. Surprisingly, the increased fertiliser use and the cost of cultivation did not improve wheat yields, which were 15% lower for rotavator adopters compared to the non‐adopters. Given the increased cost and reduced yield, it seems clear that rotavator adoption is not beneficial for farmers. The gross margin of wheat in plots with rotavator tillage was negative (a loss of NPR 2,800 or US$ 26 per hectare). In comparison, non‐adopters were making positive returns from wheat (NPR 12,200 or US$ 114 per hectare).^8^ There could be a number of factors other than tillage determining the wheat productivity and profitability, which are examined below.

**Table 1:** Wheat enterprise budgets with and without rotavator adoption in Nepal Terai

Variables	(a) Full sample (*N* = 485)		(b) Adopters (*N* = 158)		(c) Non‐adopters (*N* = 327)		Difference (%) between (b) and (c)
	Mean	SE	Mean	SE	Mean	SE	
Labour wage rate (‘000 NPR/day)	0.35	0.01	0.38	0.01	0.34	0.01	12.39***
Nitrogen (N) application rate (kg/ha)	68.44	1.45	74.29	2.27	65.59	1.82	13.27***
Phosphorus (P_2_O_5_) application rate (kg/ha)	41.83	0.84	47.30	1.38	39.18	1.02	20.71***
Tillage cost (‘000 NPR/ha)	9.15	0.16	7.98	0.24	9.72	0.20	–17.94***
Total input cost (‘000 NPR/ha)	17.63	0.37	19.77	0.76	16.60	0.40	19.12***
Total operational cost (‘000 NPR/ha)	18.66	0.33	17.72	0.61	19.11	0.39	–7.27***
Total labour cost (‘000 NPR/ha)	19.31	0.37	23.17	0.65	17.45	0.42	32.73***
Total variable cost (‘000 NPR/ha)	55.60	0.72	60.65	1.36	53.16	0.80	14.10***
Grain yield (tons/ha)	2.29	0.03	2.08	0.05	2.39	0.04	–13.12***
Gross revenue (‘000 NPR/ha)	62.91	0.93	57.83	1.36	65.36	1.19	–11.51***
Gross margin (‘000 NPR/ha)	7.31	1.02	–2.82	1.50	12.20	1.23	–123.11***

***Notes*** **:** ***Significant at 1% level. NPR stands for Nepalese Rupee (1 US$ = 107 NPR during the survey year; NRB, 2017) and SE stands for standard error of the sample mean. Material input costs include the cost of seeds, chemical fertilisers (urea, potash, DAP, other fertilisers), manures, herbicides, pesticides etc., but not depreciation cost and interest rates. Operational cost includes tillage (bullock, cultivator, rotavator), irrigation, harvesters, threshing, transport and other expenses. Labour costs include total family labour (valuated with market labour wage rate) and total hired labour. Household labour costs were valuated with opportunity cost of labour wage rate prevalence in villages. Gross revenue is estimated by multiplying total grain yield multiplied with grain price, while gross margin indicates gross revenue minus total variable cost (indicator of profit in this study).

### 5.1 Delineating the effects of rotavator tillage

To estimate farm households’ propensity to adopt rotavators, we run binary logit models using farm household and plot attributes as control variables. Table 2 shows the results for the model that includes plot‐level attributes and regional dummies. A comparison with a logit model excluding these variables (Table A2 in the online Appendix) shows that the results are relatively robust across the specifications. The majority of adopters have opportunities for non‐farm income activities, belong to one of the socially non‐marginalised castes, participate in the group activities, have access to mobile phones, and live in villages with high wage rates for agricultural labour. The logit model suggests that small farmers who rely on off‐farm income opportunities are more likely to adopt rotavator tillage. Adoption is higher in low lands and in plots with irrigation facilities. Delay in harvesting the previous crop is another important factor determining adoption, as there could be some timesaving associated with rotavator tillage.

**Table 2:** Factors affecting rotavator adoption: Logit model estimates

Variables	Coefficient	SE
Natural logarithm of farm size of the household (ha)	–0.51***	0.15
Household size (number)	0.01	0.03
Household belongs to non‐marginalised caste (1 = yes, 0 = no)	0.73***	0.23
Age of household head (years)	3E‐03	0.01
Education of household head (year)	0.01	0.03
Sex of household head (1 = male, 0 = female)	–0.11	0.38
Natural logarithm of off‐farm income (NPR/year)	–0.02	0.02
Household members migrated (number)	0.19	0.27
Groups/cooperatives membership (1 = yes, 0 = no)	0.45*	0.27
Household with mobile phones (1 = yes, 0 = no)	0.93***	0.29
Occupation of household head (1 = farming, 0 = others)	–1.28***	0.48
Labour wage rate (NPR/day)	0.01***	0.00
Land tenure (1 = if leased‐in, 0 = otherwise)	–0.50	0.32
Timely availability of fertilisers (1 = yes, 0 = no)†	0.06	0.26
Silt soil (1 = silt, 0 = others)	0.54	0.38
Clay soil (1 = clay, 0 = others)	0.25	0.41
Low land (1 = lowland, 0 = others)	0.59**	0.31
Irrigation status (1 = irrigated, 0 = not irrigated)	1.61**	0.84
Delay in harvesting previous crop (1 = yes, 0 = no)	0.70*	0.37
West (1 = if farms located in western Terai districts, 0 = others)	1.30***	0.43
Mid and far‐west (1 = if farms located in mid and far‐west Terai districts, 0 = others)	0.31	0.39
Model intercept	–6.71***	1.48
Pseudo‐*R* ^2^	0.21	
LR *χ* ^2^	127.51	
Log likelihood	–242.35	
Non‐adopters correctly predicted (%)	78.0	
Adopters correctly predicted (%)	63.0	
Model correctly predicted adopters and non‐adopters (%)	74.0	

***Notes***: ***Significant at 1% level; **Significant at 5% level; *Significant at 10% level. SE stands for standard error. Number of observations: 485.

^†^This variable indicates whether the farmer experienced any delay in obtaining fertilisers in the wheat season. Fertiliser availability in time is a persistent problem in Nepal because of the undeveloped fertiliser industry in the country.

For matching, we used three different matching algorithms to derive the treatment effect – NNM, KBM and RBM – as described in section 4. The matching procedure for each of these algorithms were checked in order to balance the distribution of observed attributes for rotavator adopters and non‐adopters. In Table A3 in the online Appendix, we present the covariates status before and after matching, which shows a substantial reduction in the percentage bias after matching. The statistical insignificance of differences in the observed attributes indicates the absence of any systematic difference between adopters and non‐adopters. The distribution of propensity scores for the rotavator adopters and non‐adopters is presented in Figure A2 (online Appendix). The overlapping of distribution of propensity scores indicates a common support for the adopter and non‐adopter sub‐samples (Rubin, 2008). The pseudo *R* ^2^ as well as the *P*‐value of the likelihood ratio became significantly lower and statistically insignificant after matching, indicating further the absence of any differences in observed attributes for these sub‐samples (Table A4 in the online Appendix). Furthermore, the mean and median bias after matching is significantly below the threshold of 20% for all the matching algorithms considered. The low bias values indicate that the balancing property is satisfied.

The results for the average treatment effect for the treated (ATT), as estimated by NNM, KBM and RBM algorithms, are presented in Table 3. Adoption of rotavator tillage indeed reduced the tillage cost for wheat farming significantly. The results are similar across different matching algorithms, and the tillage cost saved from rotavator adoption ranges between NPR 1,229 (US$ 11.5; 15.4% lower than the tillage cost of non‐adopters) and 1,586 (US$ 14.8; 20.1% lower) per hectare. There are no significant differences across the adoption categories with respect to the total variable cost; the significance of differences in the descriptive statistics vanished after matching. On the other hand, rotavator tillage is found to result in significantly lower wheat yields, ranging between 284–309 kg/ha (13.6–14.9%). As a result, the gross revenue and gross margin for wheat farming were also lower in the plots prepared with rotavators, with the reduction in gross margin of NPR 9,916–10,811 (US$ 93–101; 397–445%) per hectare.

**Table 3:** Average treatment effect on outcome variables

Matching algorithm	Outcome (per hectare)			*t*‐stat	Γ (Critical level of hidden bias)	Number of treated households	Number of control households
		ATT	SE				
Nearest neighbour matching (NNM)	Tillage cost (‘000 NPR)	–1.23***	0.45	–2.75	1.80–1.85	157	327
	Total variable cost (‘000 NPR)	1.00	2.06	0.49	–		
	Wheat yield (tons)	–0.29***	0.09	–3.03	1.85–1.90		
	Gross revenue (‘000 NPR)	–9.81***	2.77	–3.54	2.25–2.30		
	Gross margin (‘000 NPR)	–10.81***	2.92	–3.70	2.25–2.30		
Kernel based matching (KBM)	Tillage cost (‘000 NPR)	–1.59***	0.44	–3.64	2.30–2.35	143	327
	Total variable cost (‘000 NPR)	1.22	2.04	0.60	–		
	Wheat yield (tons)	–0.28***	0.09	–3.29	1.90–1.95		
	Gross revenue (‘000 NPR)	–8.69***	2.56	–3.39	2.05–2.10		
	Gross margin (‘000 NPR)	–9.92***	2.71	–3.66	2.05–2.10		
Radius based matching (RBM)	Tillage cost (‘000 NPR)	–1.48***	0.43	−3.43	2.25–2.30	143	327
	Total variable cost (‘000 NPR)	1.29	2.03	0.64	–		
	Wheat yield (tons)	–0.31***	0.08	–3.62	2.20–2.25		
	Gross revenue (‘000 NPR)	–9.31***	2.54	–3.66	2.30–2.35		
	Gross margin (‘000 NPR)	–10.61***	2.69	–3.94	2.30–2.35		

***Notes*** **:** ***Significant at 1% level. ATT, average treatment effect for the treated (adopters). SE, Standard error. Exchange rate: 1 US$ = NPR 107 (NRB, 2017).

Table 3 also includes a sensitivity analysis to detect the presence of hidden bias in the model, based on Rosenbaum bounds.^9^ The critical value of Γ ranged between Γ = 1.80–1.85 and Γ = 2.30–2.35. The value of Γ = 1.80, for example, suggests that only if two farm households with the same attributes differ in their odds of rotavator adoption by a factor of 80%, could the significance of adoption effects on yield be questioned. We can hence conclude that our estimates are robust, and the inference on the estimated effects will not change even in the presence of substantial unobserved heterogeneity.

Smith and Todd (2005) demonstrated that the treatment effects derived from matching can be sensitive to specifications of propensity scores. Following Dehejia (2005), three diagnostic tests were performed with re‐estimated treatment effects using different model specifications.^10^ Higher order covariates such as quadratic forms of farm size, household head's age, and wage rate were included in addition. In a separate model, the quantity of fertilisers and the type of seed used were included, following advice from a referee. These model estimates and sensitivity analysis of treatment effects are presented in the online Appendix (Tables A5 and A6). The treatment effects are similar to those shown in Table 3, indicating that the estimates are not affected significantly by the changes in the model specification and inclusion of additional conditioning variables.

### 5.2 Heterogeneous effects of rotavator tillage

We also examined the effects for different categories of farm households with respect to farm size, years of adoption, and sowing times. The results are shown in Table 4.

**Table 4:** Heterogeneous effects of rotavator adoption

Categories	Outcome (per hectare)	ATT	SE	*t*‐stat	Γ (Critical level of hidden bias)	Number of treated households	Number of control households
*With respect to farm size*							
Large farms (≥0.8 ha)	Wheat yield (tons)	–0.55***	0.14	–4.04	2.75–2.80	67	204
	Gross margin (‘000 NPR)	–18.42***	4.25	–4.33	3.05–3.10	67	204
Small farms (<0.8 ha)	Wheat yield (tons)	–0.11	0.11	–0.99	–	90	121
	Gross margin (‘000 NPR)	–4.63	3.29	–1.41	–	90	121
*With respect to years of adoption*							
<3 years	Wheat yield (tons)	–0.02	0.15	–0.17	–	127	58
	Gross margin (‘000 NPR)	–0.75	3.98	–0.19	–	127	58
≥3 years	Wheat yield (tons)	–0.52***	0.15	–3.36	3.25–3.30	30	269
	Gross margin (‘000 NPR)	–17.80***	4.70	–3.79	2.90–2.95	30	269
*With respect to sowing time* †							
Early sowing	Wheat yield (tons)	–0.46***	0.11	–3.20	2.60–2.65	55	134
	Gross margin (‘000 NPR)	–10.27***	3.21	–4.08	3.30–3.35	55	134
Late sowing	Wheat yield (tons)	–0.23*	0.13	–1.82	2.50–2.55	94	193
	Gross margin (‘000 NPR)	–12.53***	4.41	–2.84	1.40–1.45	94	193

***Notes*** **:** ***Significant at 1% level. *Significant at 10% level. ATT, average treatment effect for the treated (adopters). SE, Standard error. 1 US$ = 107 NPR (NRB, 2017). Matching algorithm used is NNM, in which three nearest neighbour matching with replacement and common support.

^†^Farmers sowing wheat seed on or before November 23 were considered as early sowing, and after that as late sowing.

To study the impact of rotavator tillage across different farm size categories, the sample data were stratified into large and small farms around the median value (0.8 ha). The treatment effects of rotavator tillage on wheat yield and gross margin (profit) are found to be significantly negative for larger farmers, while the effects were small and statistically insignificant for smaller farmers. For the former, the critical value gamma ranges from Γ = 2.75–2.80 for wheat yield indicating that the results will not alter even in the presence of a substantial amount of unobserved heterogeneity. The adverse effects among large farmers could be due to the early adoption of the technology compared with small farmers, and hence could be indicative of longer‐term effects (as indicated by Singh *et al*., 2013). This is further verified by stratifying the sample data into two categories, that is, <3 and ≥3 years of history of continuous adoption. The results show that farms where rotavator tillage was used continuously for ≥3 years have more pronounced negative impacts on wheat yield and farm profits. However, no significant impact was detected in the farms that had used rotavator tillage for <3 years.

In order to investigate the impact of rotavator tillage across different wheat sowing times, we stratified our data into early and late sowing groups. Our results show that wheat yield and gross margin were significantly lower for both early and late sowing groups with no significant difference between them. Although early sowing of wheat in South Asia can have a significant positive impact on wheat yield (Lobell *et al*., 2013; Keil *et al*., 2015), our results suggest that rotavator tillage could be offsetting the yield advantage of early sowing.

We also estimated the differential impacts of rotavator tillage with respect to the rate of fertiliser application, because adopters apply more fertilisers compared to non‐adopters (Table 1). These results along with the effects on soil types are presented in Table A7 (online Appendix). Among the plots with high nitrogen (N) and phosphorous (P_2_O_5_) applications, rotavator adoption led to a significant loss both in terms of grain yield and profit. When only one of the nutrients was higher, the yield and profit losses were even higher in magnitude.

## 6 Discussion and Conclusion

We examined the adoption and impacts of high‐speed rotavator tillage on wheat yield, cost of cultivation, and gross margin among lowland wheat farmers in Nepal. To the best of our knowledge, this is the first attempt to examine the effects of rotavator tillage on farmers’ fields and plots. The empirical analysis using PSM shows that, while adoption of rotavator tillage reduces the tillage cost, it also reduces wheat yield, gross revenue and farm profit. The immediate and apparent tillage cost savings from the use of rotavators appear to be perceived by farmers as more salient than the later consequences for yields and profits. We also find that small farmers are more likely to use rotavators than their larger and possibly better‐informed counterparts. Those with more off‐farm income are also more likely to use rotavators as they perhaps pay less attention to their farming practices and outcome. While adoption of new technologies is often driven by cost savings (Gitonga *et al*., 2013; Pierpaoli *et al*., 2013; Chuchird *et al*., 2017), we find that use of rotavators amongst our sample of farmers actually results in reduced yields and earnings, despite the savings in tillage costs. There is an obvious need to educate farmers regarding the negative effects of rotavator tillage, as these effects become prominent only after years of continuous use.

Although our study uses cross‐sectional data, the analysis does provide some valuable insights on long‐term adverse effects of rotavators. Farmers using rotavator tillage continuously for 3 years or more had significantly lower yields and profit than farmers who have used rotavators for less than 3 years, reflecting the longer‐term adverse effects of rotavator tillage found in field experiments. The adoption literature suggests that larger farmers tend to adopt new technology earlier (Feder and O'Mara, 1981; Feder *et al*., 1985; Rogers, 1995; Sunding and Zilberman, 2001). It is possible that large farmers in our sample have already discovered these disadvantages, and that is why they are less likely to use the technology. Although our analysis does not reveal any statistically significant difference between adopter and non‐adopters’ yields or profits for small farms, they might also suffer significant penalties in the longer‐run with continued rotavator tillage. Panel data are required to establish the changes in impact magnitude over time.

Zero tillage could be a sustainable alternative to rotavator tillage, especially in areas where human labour is scarce and the interval between two crops is short. Similar to rotavator tillage, zero tillage requires only a single machine pass, with significant time and labour saving along with yield and profit improvements being reported for zero tillage adoption (Krishna and Veettil, 2014; Keil *et al*., 2015). However, development of zero tillage needs better dissemination of information and service provision networks in many parts of South Asia, especially in the light of the conventional perceptions of the importance of a fine and clean tilth for sowing crops like wheat, as produced by the rotavator. Significant promotion programmes are required to change farmer perceptions to realise the importance of soil quality and mulching, and to prepare them to adopt more sustainable tillage practices.

Policy signals are also important. The government of Nepal has promulgated an agriculture mechanisation policy since 2014 (Gauchan and Shrestha, 2017), providing subsidies for agricultural machinery (Takeshima, 2017a), including rotavators. Our findings strongly suggest that this policy is misplaced. We suggest that a policy modification is needed to encourage farmer adoption of conservation tillage, in addition to a sustained information and benchmarking service to inform farmers of the dangers of rotavator tillage and the advantages of no‐till.

## Supporting information


**Figure A1.** Map of Nepal showing overall wheat area by district, survey location, and the spread of rotavators.
**Figure A2.** Distribution and common support for propensity score.
**Table A1.** Socio‐economic characteristics of rotavator adopters and non‐adopters in Nepal Terai.
**Table A2.** Factors affecting rotavator adoption (excluding plot level attributes): Logit model estimates.
**Table A3.** Test for selection bias after matching.
**Table A4.** Statistical test to evaluate bias‐reduction after matching.
**Table A5.** Logit model estimates for sensitivity analysis.
**Table A6.** Average treatment effects for rotavator adopters under different specifications of selection model.
**Table A7.** Heterogeneous effects of rotavator adoption across soil types and fertiliser application rates.

## Notes

2 Out of the 75 districts of Nepal, 20 lie in the Terai region (MoAD, 2016). The selected 10 districts were Kanchanpur, Kailali, Bardyia, Banke, Rupendhai, Nawalparsi, Bara, Dhanusa, Saptari and Sunsari.

3 The sample households managing more than one wheat plot were asked to provide inputs and output from their main plot. These households may use different levels of inputs in different plots, and these plots may differ with respect to soil type and topography. Eliciting details of different attributes of all plots managed by the household would have increased the survey time and cost significantly, and would have reduced the data quality.

4 The other tillage methods in this study include only conventional practices. None of the sample farmers has adopted zero tillage.

5 Finding an ideal instrument for a cross‐sectional impact study is a difficult task (Angrist and Pischke, 2009). We have conducted a detailed examination of the recent adoption‐impact literature for a suitable instrument. Some of the variables used as instruments in the past studies (e.g. group membership) did not satisfy the exclusion restriction. Some other potential candidates, (e.g. year of introduction of rotavators in the village) were also found to be unsuitable as an instrument when tested using an approach suggested by Di Falco *et al*. (2011).

6 However, since we have purposively sampled the districts and VDCs based on wheat acreage and prevalence of mechanised tillage, these adoption rates may not be representative of the study region.

7 Among the four major caste categories prevalent in Nepal (Brahmin, Chettri, Vaish and Shudra), Vaish and Shudra are considered as the socially marginalised castes.

8 Use of gross margin to capture economic superiority of a technology is criticised in the literature, as the estimation does not capture the opportunity cost of family labour time. If farmers save time through the use of rotavators and they have a high opportunity cost of time, then it is economically rational for them to adopt in spite of lower yields and gross margins. However, opportunity costs are difficult to estimate, being dependent on a multitude of factors. In this study, we have taken annual off‐farm income as the proxy variable; off‐farm income of adopters (152,000 NPR) is not significantly different when compared with that of non‐adopters (156,000 NPR). That is, inclusion of opportunity cost of family labour may not drastically alter our finding on the economic unsustainability of the rotavator technology.

9 Since sensitivity analysis of insignificant variables is meaningless (Hujer *et al*., 2004), Rosenbaum bounds were derived only for the variables that are statistically significant.

10 By adding fertiliser use and improved variety adoption as conditioning variables in the logit model, we re‐estimated the treatment effect and the results are shown in specification‐3 in Tables A5 and A6 in the online Appendix.
