# Supplementary material for: Apparent Gains, Hidden Costs: Examining Adoption Drivers, Yield, and Profitability Outcomes of Rotavator Tillage in Wheat Systems in Nepal
Source: J Agric Econ. 2019 Apr 29;71(1):199–218. doi: 10.1111/1477-9552.12333 (PMC6988502; doi:10.1111/1477-9552.12333)
Supplement: Supplementary file 1 — Figure A1. Map of Nepal showing overall wheat area by district, survey location, and the spread of rotavators. Figure A2. Distribution and common support for propensity score. Table A1. Socio‐economic characteristics of rotavator adopters and non‐adopters in Nepal Terai. Table A2. Factors affecting rotavator adoption (excluding plot level attributes): Logit model estimates. Table A3. Test for selection bias after matching. Table A4. Statistical test to evaluate bias‐reduction after matching. Table A5. Logit model estimates for sensitivity analysis. Table A6. Average treatment effects for rotavator adopters under different specifications of selection model. Table A7. Heterogeneous effects of rotavator adoption across soil types and fertiliser application rates. [file JAGE-71-199-s001.docx]

**Apparent Gains, Hidden Costs: Examining Adoption Drivers, Yield and Profitability Outcomes of Rotavator Tillage in Wheat Systems in Nepal**

Gokul P. Paudel, Vijesh V. Krishna and Andrew J. McDonald

*Journal of Agricultural Economics*

**Online Appendix**

Figure A1: Map of Nepal showing overall wheat area by district, survey location, and the spread of rotavators


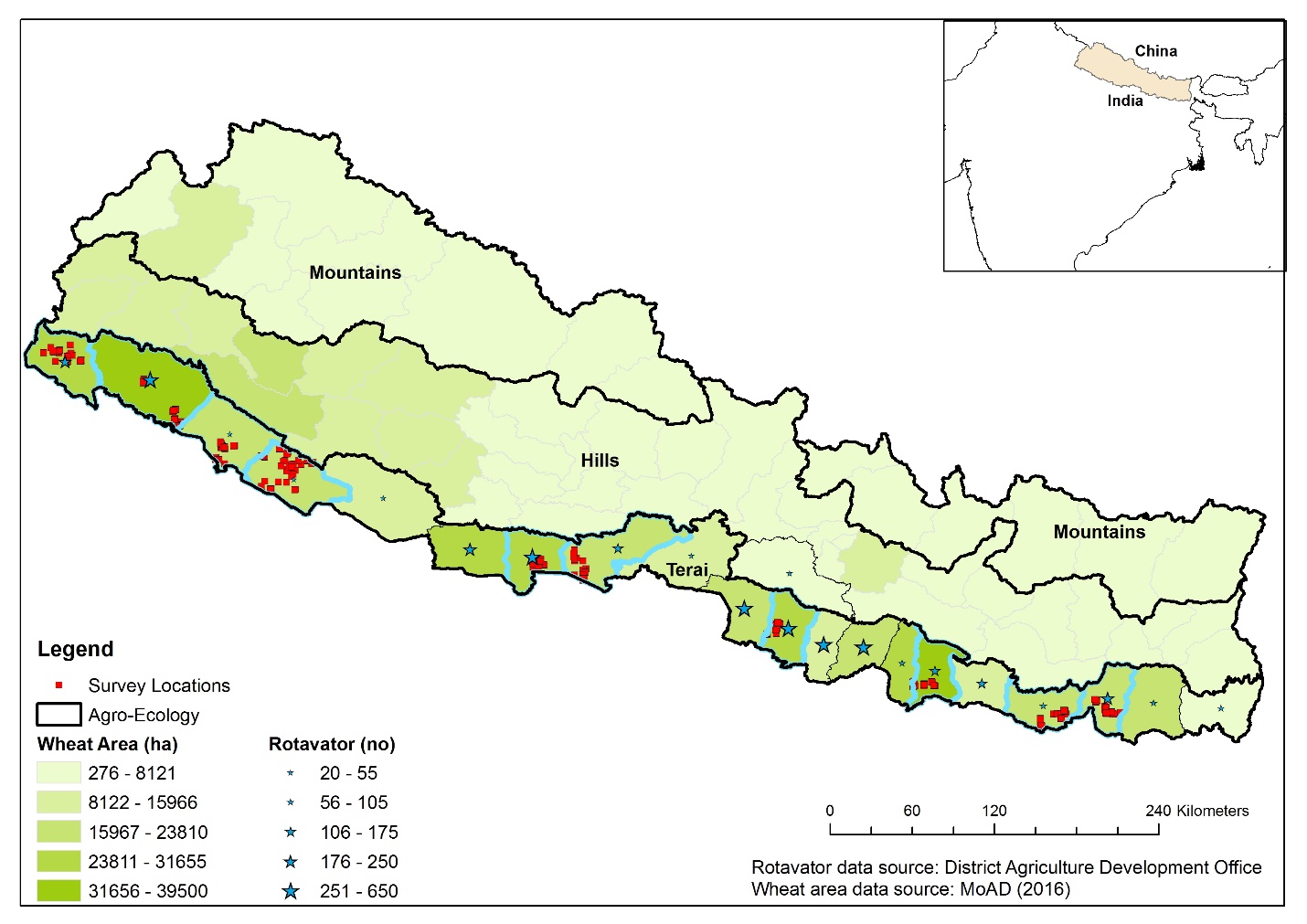


Figure A2: Distribution and common support for propensity score

***Note***: "Treated: On support” category includes rotavator adopters with suitable matching pairs. “Treated: Off support” includes rotavator adopters for whom matching pairs could not be found.

Table A1

Socio-economic characteristics of rotavator adopters and non-adopters in Nepal Terai

|  | (a) Full sample (N=485) | | (b) Adopters  (N=158) | | (c) Non-adopters  (N=327) | | Difference (%) between  (b) and (c) |
| --- | --- | --- | --- | --- | --- | --- | --- |
|  | Mean | SE | Mean | SE | Mean | SE |  |
| *Household characteristics* |  |  |  |  |  |  |  |
| Farm size of the household (ha) | 1.27 | 0.11 | 0.95 | 0.09 | 1.42 | 0.15 | --33.15*** |
| Household size (number) | 7.17 | 0.20 | 7.57 | 0.45 | 6.98 | 0.20 | 8.37 |
| Household belongs to a non-marginalised caste (1=yes, 0=no) | 0.46 |  | 0.56 |  | 0.41 |  | 34.87*** |
| Age of household head (years) | 47.18 | 0.47 | 47.37 | 0.83 | 47.09 | 0.57 | 0.61 |
| Education of household head (years in school) | 5.99 | 0.18 | 6.21 | 0.32 | 5.88 | 0.21 | 5.68 |
| Sex of household head (1=male, 0=female) | 0.88 |  | 0.88 |  | 0.89 |  | --0.79 |
| Off farm income (‘000 NPR/year) | 154.41 | 6.94 | 151.68 | 10.88 | 155.73 | 8.86 | --2.60 |
| Household members migrated (number) | 0.33 | 0.02 | 0.32 | 0.04 | 0.33 | 0.03 | --5.10 |
| Group / cooperative membership (1=yes, 0=no) | 0.53 |  | 0.58 |  | 0.51 |  | 14.57 |
| Household with mobile phones (1=yes, 0=no) | 0.42 |  | 0.52 |  | 0.37 |  | 40.75*** |
| Occupation of household head (1=farming, 0=others) | 0.94 |  | 0.89 |  | 0.97 |  | --7.66*** |
| Land tenure (1=if leased-in, 0=otherwise) | 0.22 |  | 0.18 |  | 0.24 |  | -24.90 |
| West (1= if farms located in western Terai districts, 0=others) | 0.21 |  | 0.31 |  | 0.16 |  | 98.72*** |
| Mid and far-west (1= if farms located in mid and far-west Terai districts, 0=others) | 0.41 |  | 0.33 |  | 0.44 |  | --23.86** |
| *Plot characteristics* |  |  |  |  |  |  |  |
| Timely availability of fertilisers (1=yes, 0=no) | 0.66 |  | 0.65 |  | 0.66 |  | --2.71 |
| Silt soil (1=silt, 0=others) | 0.57 |  | 0.65 |  | 0.53 |  | 22.56*** |
| Clay soil (1=clay, 0=others) | 0.23 |  | 0.26 |  | 0.22 |  | 16.14 |
| Low land (1=lowland, 0=others) | 0.20 |  | 0.27 |  | 0.17 |  | 55.56*** |
| Irrigation status (1=irrigated, 0=not irrigated) | 0.95 |  | 0.99 |  | 0.94 |  | 5.45*** |
| Delay in harvesting previous crop (1=yes, 0=no) | 0.19 |  | 0.28 |  | 0.15 |  | 93.88*** |
| Sowing time in Julian days of year (1=before Nov 23, 0=after Nov 23) | 334.55 |  | 336.30 |  | 333.71 |  | 0.78** |

***Notes***: *** Significant at 1% level; ** Significant at 5% level. SE stands for standard error of sample mean. NPR stands for Nepalese Rupee (1 US$ = 107 NPR during 2016, the survey year (NRB, 2017)).

Table A2

Factors affecting rotavator adoption (excluding plot level attributes): Logit model estimates

| Variables | Coefficient | SE |
| --- | --- | --- |
| Natural logarithm of farm size of the household (ha) | -0.58*** | 0.14 |
| Household size (number) | 0.04 | 0.03 |
| Household belongs to a non-marginalised caste (1=yes, 0=no) | 0.60*** | 0.22 |
| Age of household head (years) | 3E-03 | 0.01 |
| Education of household head (year) | 3E-03 | 0.03 |
| Sex of household head (1=male, 0=female) | 0.08 | 0.36 |
| Natural logarithm of off-farm income (NPR/year) | -0.02 | 0.02 |
| Household members migrated (number) | -0.01 | 0.25 |
| Group /cooperatives membership (1=yes, 0=no) | 0.34 | 0.23 |
| Household with mobile phones (1=yes, 0=no) | 0.90*** | 0.24 |
| Occupation of household head (1=farming, 0=others) | -1.18** | 0.47 |
| Labor wage rate (NPR/day) | 0.01*** | 0.00 |
| Land tenure (1=if leased-in, 0=otherwise) | -0.66** | 0.31 |
| Timely availability of fertilisers (1=yes, 0=no)^#^ | -0.07 | 0.24 |
| Model intercept | -5.54*** | 1.13 |
| Pseudo-*R*^2^ | 0.17 |  |
| LR *χ*^2^ | 102.80 |  |
| Log likelihood | -254.7 |  |
| Non-adopters correctly predicted (%) | 72.9 |  |
| Adopters correctly predicted (%) | 63.0 |  |
| Model correctly predicted adopters and non-adopters (%) | 72.9 |  |

***Notes***: *** Significant at 1% level; ** Significant at 5% level. SE stands for standard error. Number of observations: 485.

Table A3

Test for selection bias after matching

| Variable | Matched samples | | % Bias | % Bias reduction | *P*-value from *t*-test |
| --- | --- | --- | --- | --- | --- |
|  | Treated | Control |  |  |  |
| Farm size of the household (ha) | 0.95 | 1.09 | -6.30 | 71.70 | 0.36 |
| Household size (number) | 7.49 | 7.65 | -3.30 | 73.10 | 0.77 |
| Household belongs to a non-marginalised caste (1= yes, 0=no) | 0.55 | 0.51 | 8.10 | 72.00 | 0.48 |
| Age of household head (years) | 47.54 | 48.43 | -8.60 | -213.20 | 0.45 |
| Education of household head (years in school) | 6.20 | 6.18 | 0.50 | 93.70 | 0.96 |
| Sex of household head (1=male, 0=female) | 0.88 | 0.90 | -5.90 | -169.00 | 0.59 |
| Off farm income (‘000 NPR/year) | 150.00 | 160.00 | -8.90 | -226.80 | 0.43 |
| Household members migrated (number) | 0.32 | 0.33 | -3.20 | 11.90 | 0.78 |
| Group / cooperative membership (1=yes, 0=no) | 0.59 | 0.58 | 0.90 | 94.30 | 0.94 |
| Household with mobile phones (1=yes, 0=no) | 0.52 | 0.59 | -14.20 | 54.00 | 0.21 |
| Occupation of household head (1=farming, 0=others) | 0.89 | 0.88 | 8.40 | 71.30 | 0.55 |
| Labor wage rate (‘000 NPR/day) | 0.38 | 0.01 | 3.00 | 95.70 | 0.80 |
| Land tenure (1=if leased-in, 0=otherwise) | 0.18 | 0.14 | 9.80 | 34.00 | 0.33 |
| Timely availability of fertilisers (1=yes, 0=no) | 0.64 | 0.65 | -2.20 | 41.20 | 0.84 |
| Silt soil (1=silt, 0=others) | 0.65 | 0.61 | 8.30 | 66.30 | 0.46 |
| Clay soil (1=clay, 0=others) | 0.26 | 0.29 | -6.90 | 18.00 | 0.56 |
| Low land (1=lowland, 0=others) | 0.26 | 0.27 | -1.00 | 95.50 | 0.93 |
| Irrigation status (1=irrigated, 0=not irrigated) | 0.99 | 0.98 | 5.60 | 79.40 | 0.48 |
| Delay in harvesting previous crop (1=yes, 0=no) | 0.28 | 0.32 | -8.90 | 73.80 | 0.49 |
| Western (1= if farms located in western Terai districts, 0=others) | 0.34 | 0.37 | -6.10 | 71.70 | 0.58 |
| Mid and far-west (1= if farms located in mid and far-west Terai districts, 0=others) | 0.31 | 0.28 | 6.60 | 82.10 | 0.59 |

Table A4

Statistical test to evaluate bias-reduction after matching

| Matching method | Pseudo *R*^2^ | Likelihood ratio *χ*^2^ | *P*>*χ*^2^ | Mean bias | Median bias |
| --- | --- | --- | --- | --- | --- |
| Before matching | 0.21 | 127.50 | 0.00 | 20.7 | 21.9 |
| Nearest neighbour matching (NNM) | 0.02 | 7.68 | 0.99 | 5.5 | 6.0 |
| Kernel based matching (KBM) | 0.02 | 7.28 | 0.99 | 5.9 | 5.1 |
| Radius based matching (RBM) | 0.02 | 6.52 | 0.99 | 5.4 | 5.3 |

Table A5

Logit model estimates for sensitivity analysis

| Variables | Specification-1 | | Specification-2 | | Specification-3 | |
| --- | --- | --- | --- | --- | --- | --- |
|  | Coefficient | SE | Coefficient | SE | Coefficient | SE |
| Natural logarithm of farm size of the household (ha) | -0.50*** | 0.15 | -0.48*** | 0.16 | -0.60*** | 0.15 |
| Household size (number) | -1E-03 | 0.01 | 0.01 | 0.03 | 0.01 | 0.03 |
| Household belongs to a non-marginalised caste (1=yes, 0=no) | 0.01*** | 0.09 | 0.73*** | 0.23 | 0.71*** | 0.24 |
| Age of household head (years) | -0.01 | 0.37 | 0.01 | 0.07 | 3E-03 | 0.01 |
| Education of household head (year) | 0.67 | 0.23 | 0.01 | 0.03 | 0.02 | 0.03 |
| Sex of household head (1=male, 0=female) | 0.03 | 0.03 | -0.09 | 0.38 | -0.06 | 0.38 |
| Natural logarithm of off-farm income (NPR/year) | -0.02 | 0.02 | -0.02 | 0.02 | -0.02 | 0.02 |
| Household members migrated (number) | 0.05 | 0.26 | 0.22 | 0.27 | 0.27 | 0.28 |
| Group / cooperative membership (1=yes, 0=no) | 0.49** | 0.24 | 0.45* | 0.27 | 0.55** | 0.28 |
| Household with mobile phones (1=yes, 0=no) | 0.66*** | 0.27 | 0.94*** | 0.30 | 0.80*** | 0.30 |
| Occupation of household head (1=farming, 0=others) | -1.26** | 0.49 | -1.34*** | 0.49 | -1.53*** | 0.49 |
| Labour wage rate (NPR/day) | 0.01 | 0.02 | 0.02 | 0.02 | 0.01*** | 0.00 |
| Land tenure (1=if leased-in, 0=otherwise) | -0.62* | 0.32 | -0.48 | 0.33 | -0.51 | 0.34 |
| Timely availability of fertilisers (1=yes, 0=no) | -0.06 | 0.25 | 0.07 | 0.26 | -0.04 | 0.26 |
| Silty soil (1=silty, 0=others) | 0.71* | 0.36 | 0.54 | 0.38 | 0.44 | 0.39 |
| Clayey soil (1=clayey, 0=others) | 0.31 | 0.41 | 0.24 | 0.42 | 0.14 | 0.42 |
| Low land (1=lowland, 0=others) | 0.50* | 0.31 | 0.58* | 0.31 | 0.61** | 0.32 |
| Irrigation status (1=irrigated, 0=not irrigated) | 1.59* | 0.82 | 1.58* | 0.83 | 1.74** | 0.86 |
| Delay in harvesting previous crop (1=yes, 0=no) | 0.43 | 0.32 | 0.71* | 0.37 | 0.73** | 0.38 |
| West (1= if farms located in western Terai districts, 0=others) | – | – | 1.39*** | 0.45 | 1.10*** | 0.44 |
| Mid and far-west (1= if farms located in mid and far-west Terai districts, 0=others) | – | – | 0.34 | 0.40 | 0.45 | 0.41 |
| Farm size × farm size | -3E-03 | 0.01 | -3E-03 | 0.01 | – | – |
| Age × age | -1E-03 | 0.01 | -1.E-04 | 8E-04 | – | – |
| Wage rate × wage rate | 9E-06 | 2E-05 | -2E-05* | 3E-05 | – | – |
| Wheat variety (1=improved, 0=others) | – | – | – | – | 0.57 | 0.40 |
| Nitrogen applied (kg/ha) | – | – | – | – | -9E-05 | 0.01 |
| Phosphorus applied (kg/ha) | – | – | – | – | 0.02*** | 0.01 |
| Model intercept | -5.75* | 3.44 | -9.38** | 3.92 | -8.19*** | 1.58 |
| Pseudo-*R*^2^ | 0.19 |  | 0.21 |  | 0.23 |  |
| LR *χ*^2^ | 117.61 |  | 128.37 |  | 142.60 |  |
| Log likelihood | -247.30 |  | -241.92 |  | -234.80 |  |
| Non-adopters correctly predicted (%) | 77.84 |  | 77.41 |  | 79.05 |  |
| Adopters correctly predicted (%) | 66.09 |  | 62.3 |  | 65.35 |  |
| Model correctly predicted adopters and non-adopters (%) | 75.05 |  | 73.61 |  | 75.46 |  |

***Notes***: *** Significant at 1% level; ** Significant at 5% level; * Significant at 10% level. SE stands for standard error. Number of observations: 485.

Table A6

Average treatment effects for rotavator adopters under different specifications of selection model

| Specification of logit model (from Table A5) | Outcome variable (per hectare) | ATT  (SE) | Pseudo-*R*^2^ | | Likelihood ratio *χ*^2^ | | Mean standardised bias | | % bias reduction | $\Gamma$ (Critical level  of hidden bias) |
| --- | --- | --- | --- | --- | --- | --- | --- | --- | --- | --- |
|  |  |  | before matching | after matching | before matching | after matching | before matching | after matching |  |  |
| Specification-1 | Tillage cost (‘000 NPR) | -1.40***  (0.41) | 0.22 | 0.04 | 133.61***  (*P*=0.00) | 19.65  (*P*=0.66) | 22.3 | 5.6 | 74.89 | 2.30 – 2.35 |
|  | Gross revenue (‘000 NPR) | -8.31***  (2.38) | 0.23 | 0.05 | 143.48***  (*P*=0.00) | 23.09  (*P*=0.46) | 21.8 | 5.7 | 73.85 | 2.35 – 2.40 |
|  | Gross margin (‘000 NPR) | -11.13***  (2.53) | 0.24 | 0.07 | 149.17***  (*P*=0.00) | 30.10  (*P*=0.15) | 23.3 | 6.2 | 73.39 | 2.85 – 2.90 |
|  | Wheat yield (tons) | -0.27***  (0.08) | 0.23 | 0.05 | 140.40***  (*P*=0.00) | 19.87  (*P*=0.65) | 22.2 | 5.6 | 74.77 | 2.10 – 2.15 |
|  | Total variable cost (‘000 NPR) | 2.81  (1.91) | 0.19 | 0.02 | 119.43***  (*P*=0.00) | 7.00  (*P*=0.99) | 22.2 | 4.6 | 79.28 | – |
| Specification-2 | Tillage cost (‘000 NPR) | -1.21***  (0.42) | 0.24 | 0.04 | 144.53***  (*P*=0.00) | 15.97  (*P*=0.92) | 22.9 | 5.7 | 75.11 | 1.95 – 2.00 |
|  | Gross revenue (‘000 NPR) | -9.14***  (2.43) | 0.25 | 0.06 | 155.44***  (*P*=0.00) | 26.26  (*P*=0.39) | 22.4 | 6.1 | 72.77 | 2.45 – 2.50 |
|  | Gross margin (‘000 NPR) | -10.65***  (2.58) | 0.26 | 0.07 | 161.05***  (*P*=0.00) | 30.38  (*P*=0.21) | 23.7 | 6.3 | 73.42 | 2.65 – 2.70 |
|  | Wheat yield (tons) | -0.27***  (0.08) | 0.25 | 0.05 | 154.55***  (*P*=0.00) | 23.65  (*P*=0.54) | 22.8 | 5.9 | 74.12 | 2.10 – 2.15 |
|  | Total variable cost (‘000 NPR) | 1.51  (1.94) | 0.21 | 0.01 | 130.79***  (*P*=0.00) | 4.70  (*P*=0.99) | 22.7 | 4.6 | 79.74 | – |
| Specification-3 | Tillage cost (‘000 NPR) | -1.10***  (0.44) | 0.26 | 0.04 | 160.64***  (*P*=0.00) | 13.24  (*P*=0.97) | 23.2 | 4.4 | 81.03 | 1.65 – 1.70 |
|  | Gross revenue (‘000 NPR) | -10.06***  (2.57) | 0.29 | 0.08 | 175.81***  (*P*=0.00) | 29.46  (*P*=0.24) | 22.7 | 5.2 | 77.09 | 2.35 – 2.40 |
|  | Gross margin (‘000 NPR) | -10.41***  (2.75) | 0.28 | 0.06 | 169.01***  (*P*=0.00) | 23.89  (*P*=0.53) | 24.0 | 5.1 | 78.75 | 2.25 – 2.30 |
|  | Wheat yield (tons) | -0.35***  (0.09) | 0.28 | 0.08 | 174.35***  (P=0.00) | 29.32  (*P*=0.25) | 23.0 | 5.3 | 76.95 | 2.50 – 2.55 |
|  | Total variable cost (‘000 NPR) | 0.35  (2.07) | 0.23 | 0.01 | 143.66***  (*P*=0.00) | 3.97  (*P*=0.99) | 23.0 | 3.2 | 86.08 | – |

***Notes***: *** Significant at 1% level. ATT: Average treatment effect for the treated (adopters). SE: Standard error. NPR stands for Nepalese Rupee (1 US$ = 107 NPR during 2016, the survey year; NRB, 2017). Matching algorithm used is Kernel based matching with band width 0.01 and common support.

Table A7

Heterogeneous effects of rotavator adoption across soil types and fertiliser application rates

| Categories | Outcome variable (per hectare) | ATT | SE | *t*-stat | $\Gamma$ (Critical level  of hidden bias) | Number of treated households | Number of control households |
| --- | --- | --- | --- | --- | --- | --- | --- |
| *With respect to soil type^#^* | | | | | | | |
| Silt | Wheat yield (tons) | -0.20* | 0.13 | -1.68 | 1.25 – 1.30 | 102 | 174 |
|  | Gross margin (‘000 NPR) | -8.68** | 3.80 | -2.29 | 1.70 – 1.75 | 102 | 174 |
| Clay | Wheat yield (tons) | -0.27** | 0.13 | -2.03 | 2.20 – 2.25 | 41 | 73 |
|  | Gross margin (‘000 NPR) | -9.09* | 5.10 | -1.78 | 1.75 – 1.80 | 41 | 73 |
| *With respect to fertiliser application^##^* | | | | | | | |
| Limited dose N and P_2_O_5_ | Wheat yield (tons) | -0.18 | 0.12 | -1.54 | – | 63 | 203 |
|  | Gross margin (‘000 NPR) | -7.76** | 3.82 | -2.03 | 1.60 – 1.65 | 63 | 203 |
| High P_2_O_5_ and limited N | Wheat yield (tons) | -0.55*** | 0.19 | -2.87 | 3.90 – 3.95 | 26 | 33 |
|  | Gross margin (‘000 NPR) | -17.48*** | 4.84 | -3.61 | 4.80 – 4.75 | 26 | 33 |
| High N and limited P_2_O_5_ | Wheat yield (tons) | -0.66** | 0.33 | -1.99 | 3.80 – 3.85 | 16 | 37 |
|  | Gross margin (‘000 NPR) | -22.22* | 11.43 | -1.94 | 3.80 – 3.85 | 16 | 37 |
| Both N and P_2_O_5_ not limited | Wheat yield (tons) | -0.30* | 0.16 | -1.95 | 2.20 – 2.25 | 39 | 61 |
|  | Gross margin (‘000 NPR) | -9.02* | 4.73 | -1.91 | 2.10 – 2.15 | 39 | 61 |

***Notes***: *** Significant at 1% level. ** Significant at 5% level. ATT: Average treatment effect for the treated (adopters). SE: Standard error. 1 US$ = 107 Nepalese Rupees: NPR (NRB, 2017). Matching algorithm used is NNM, in which three nearest neighbor matching with replacement and common support.

*^#^* Estimation was not carried out for sandy soils as adoption rate was marginal.

*^##^* Farms applying more than 75^th^ percentile of N (≥ 80 kg/ha) and P_2_O_5_ (≥ 50 kg/ha) were considered as not limited dose while those applying less than that were considered as limited dose of N and P_2_O_5._

Table A7 presents the heterogeneous effects of rotavator adoption across different rates of fertiliser application. The results show that, among the plots where nitrogen (N) and phosphorous (P_2_O_5_) applications were high, rotavator adoption leads to a greater loss both in terms of grain yield and profit. More strikingly, when only one of the nutrients was higher, the yield and profit losses due to rotavator tillage adoption were even higher. Among the plots where both nutrients were limiting, the loss of grain yield was not significant and the reduction in gross margins was less pronounced. These results suggest that the effects of rotavators depend heavily on the level of fertilisers applied. A review of related literature also indicates that the higher level of fertiliser application in plots with rotavator tillage aggravates the lodging problem due to shallow tillage and soil compaction by impeding root growth, nitrogen leaching and volatilisation (Majeed et al., 2015; Guan et al., 2015; Głab, 2014; Izumi et al., 2004; Bennie and Botha, 1986). No significant impact heterogeneity was detected across different soil types.

**References in the Online Appendix**

Bennie, A. T. P. and Botha, F. J. P. ‘Effect of deep tillage and controlled traffic on root growth, water-use efficiency and yield of irrigated maize and wheat’, *Soil and Tillage Research*, Vol. 7, (1986) pp. 85–95.

Głab, T. ‘Effect of soil compaction and N fertilization on soil pore characteristics and physical quality of sandy loam soil under red clover/grass sward’, *Soil and Tillage Research*, Vol. 144, (2014) pp. 8–19.

Guan, D., Zhang, Y., Al-kaisi, M. M., Wang, Q. and Zhang, M. ‘Tillage practices effect on root distribution and water use efficiency of winter wheat under rain-fed condition in the North China Plain’, *Soil & Tillage Research*, Vol. 146, (2015) pp. 286–295.

Izumi, Y., Uchida, K. and Iijima, M. ‘Crop production in successive wheat-soybean rotation with no-tillage practice in relation to the root system development’, *Plant Production Science*, Vol. 7, (2004) pp. 329–336.

Majeed, A., Muhmood, A., Niaz, A., Javid, S., Ahmad, Z. A., Shah, S. S. H. and Shah, A. H. ‘Bed planting of wheat (Triticum aestivum L.) improves nitrogen use efficiency and grain yield compared to flat planting’, *Crop Journal*, Vol. 3, (2015) pp. 118–124.

NRB. Nepal Rastra Bank 2017. Available online at: https://nrb.org.np/fxmexchangerate.php (last accessed 11 December 2017).
